# Anterior–posterior versus posterior-only stabilization of unstable pelvic ring injuries in polytraumatized patients: a comparative analysis of clinical, radiological, and long-term functional outcomes

**DOI:** 10.1007/s00264-026-06966-1

**Published:** 2026-07-20

**Authors:** Tim Westenhöfer, Hatem Alabdulrahman, Felix M. Bläsius, Rald V. M. Groven, Till Berk, Frank Hildebrand, Klemens Horst, Eftychios Bolierakis

**Affiliations:** 1 Department of Orthopaedics, Trauma and Reconstructive Surgery, University Hospital RWTH Aachen, Aachen, Germany; 2 Department of Trauma, Hand- and Reconstructive Surgery, University Hospital Münster, Münster, Germany

**Keywords:** Polytrauma, Pelvic ring fracture, Osteosynthesis, Functional outcome

## Abstract

**Background:**

Pelvic ring fractures (PRF) in polytraumatized patients can have a substantial impact on both short- and long-term outcomes. This study aimed to compare the outcomes of combined anterior–posterior (AP) stabilization versus posterior-only (PO) fixation for unstable PRFs in polytraumatized patients.

**Methods:**

This retrospective, cross-sectional cohort study included adult polytraumatized patients with AO/OTA type B and C pelvic ring injuries treated at a Level I trauma centre between 2010 and 2023. Patients were assigned to one of two groups according to the definitive pelvic stabilization strategy: posterior-only (PO) fixation or combined anterior–posterior (AP) stabilization. The primary outcomes were functional recovery and quality of life, assessed using the Pelvic Outcome Score and the EuroQol EQ-5D-5L. Secondary outcomes included complication rates, quality of radiographic fracture reduction, time to mobilization, length of hospital stay, and pain at discharge.

**Results:**

A total of 158 patients were included in the study; most were male (*n* = 102, 64.6%), with a median age of 42 years. Fifty-eight patients (36.7%) completed the cross-sectional follow-up assessment after a median follow-up of four years. Overall, 31.0% (*n* = 49) underwent definitive posterior-only (PO) stabilization, whereas 69.0% (*n* = 109) underwent combined anterior–posterior (AP) stabilization according to the institutional treatment protocol. AO/OTA type C injuries were significantly more common in the AP group. No significant differences were observed between the AP and PO groups with respect to length of hospital stay, complication rates, time to mobilization, functional outcomes, or quality of life. AP stabilization was associated with superior radiographic fracture reduction, particularly of the anterior pelvic ring, but this did not translate into improved long-term outcomes. PO fixation was associated with lower pain scores at follow-up (median VAS, 2 vs. 4; *p* < 0.05), particularly among patients with AO/OTA type B injuries.

**Conclusions:**

Both AP and PO stabilization, when selected according to the institutional treatment protocol, yielded comparable functional outcomes and complication rates in polytraumatized patients with unstable pelvic ring fractures. The choice of stabilization strategy should be individualized based on pelvic injury morphology, patient physiology, and overall trauma burden within the framework of Early Appropriate Care principles. Further prospective studies are needed to validate these findings and identify factors influencing long-term recovery.

**Supplementary information:**

The online version contains supplementary material available at https://doi.org/10.1007/s00264-026-06966-1.

## Background

Pelvic ring fractures (PRF) account for only 3–6% of all fractures, with an incidence of up to 77 per 100,000 population annually. However, the severity of the pelvic injury and the chosen treatment strategy have a substantial impact on both short- and long-term outcomes [1–3]. In polytraumatized patients, PRFs occur in up to 20% of cases and significantly contribute to morbidity and mortality [4, 5]. Unstable pelvic ring injuries, classified as AO/OTA type B or C (Arbeitsgemeinschaft für Osteosynthesefragen/Orthopaedic Trauma Association classification), are frequently associated with haemorrhagic shock and concomitant head or abdominal injuries [6]. Furthermore, nearly one-third of survivors experience persistent pain and functional limitations in activities of daily living [7].

Following temporary stabilization and patient resuscitation, the choice of definitive osteosynthetic fixation influences fracture healing and may have important implications for functional recovery [7, 8]. However, it remains unclear whether unstable pelvic ring injuries involving both the anterior and posterior pelvic ring require combined anterior–posterior (AP) stabilization or whether posterior-only (PO) fixation provides comparable outcomes while reducing the operative burden [5, 9–12]. Some authors have suggested that combined AP stabilization of lateral compression pelvic ring injuries may improve pain and facilitate earlier mobilization compared with a posterior-only approach [13, 14]. In contrast, Moussa et al. reported no significant differences in functional outcomes or complication rates between AP and PO stabilization [15]. Similarly, Zhang et al. found that PO fixation was associated with functional outcomes comparable to those of combined AP fixation, as assessed using the Majeed score [16].

Given these conflicting findings, no consensus has been reached regarding the optimal definitive stabilization strategy for unstable pelvic ring fractures, particularly in polytraumatized patients. At our institution, the decision to perform isolated posterior-only (PO) fixation or combined anterior–posterior (AP) stabilization is guided by a standardized treatment algorithm. Accordingly, the aim of this study was to compare the short- and long-term outcomes of AP versus PO stabilization in polytraumatized patients with unstable pelvic ring fractures treated according to this protocol. We hypothesized that protocol-driven patient selection for AP or PO stabilization would result in comparable functional and clinical outcomes despite differences in injury severity between the treatment groups.

## Methods

### Ethical considerations

This retrospective, cross-sectional cohort study was approved by the local institutional review board (Ethics Committee at the RWTH Aachen Faculty of Medicine, No. EK 23–372). Written consent for data processing was obtained from all patients during in-hospital treatment and at follow-up. The study was conducted in accordance with the Declaration of Helsinki and Good Clinical Practice guidelines and is reported in accordance with the Strengthening the Reporting of Observational Studies in Epidemiology (STROBE) statement [17]. The study was registered ad hoc in the German Clinical Trials Register (Deutsches Register Klinischer Studien, DRKS00033420).

### Study population

#### Inclusion criteria

Adult polytraumatized patients (≥ 18 years of age) with AO/OTA type B and C pelvic ring injuries treated at a Level I trauma centre between 2010 and 2023 were identified through administrative database screening using codes from the 10th revision of the International Classification of Diseases (ICD-10): S32.7, S32.89, S32.1, S32.3, S32.5, S33.2, S33.3, S33.4, M25.35, M53.3). Polytrauma was defined according to the Berlin Polytrauma Definition [18].

#### Exclusion criteria

Patients with concomitant acetabular fractures, death prior to surgical intervention, or lack of consent for data processing were excluded from the analysis.

#### Study cohorts

Patients who underwent isolated posterior pelvic ring fixation for definitive treatment were assigned to the posterior-only (PO) group. Patients who received stabilization of both the anterior and posterior pelvic ring were assigned to the combined anterior–posterior (AP) group. According to the institutional standard operating procedures, the decision to proceed with either emergency external fixation followed by delayed definitive stabilization or early definitive osteosynthesis was guided by the patient’s trauma burden and physiological status, in accordance with the principles of Early Appropriate Care (EAC) [19, 20]. Regarding definitive management, in cases of complete disruption of the posterior pelvic ring, osteosynthetic stabilization was performed in all cases to facilitate earlier mobilization, as supported by clinical and biomechanical evidence [21–23]. For the anterior pelvic ring, following stabilization of the posterior pelvis, surgical intervention was indicated in cases of dynamic pubic symphysis diastasis greater than 25 mm, as assessed under anesthesia [24]. For pubic rami fractures, osteosynthetic stabilization was indicated in cases of static or dynamic displacement exceeding 5 mm on compression stress testing or in the presence of comminution or fracture obliquity, as previously described in an institutional publication [13]. Percutaneous techniques were preferred over open reduction in fracture patterns amenable to closed reduction.

### Data extraction

The following data were extracted through retrospective review of patient records: age (years), sex, AO/OTA classification [25], presence of complex pelvic trauma (defined as pelvic ring fracture associated with pelvic soft-tissue injury) [26], in-hospital mortality, length (LOS) of hospital and intensive care unit (ICU) stay (days), Injury Severity Score (ISS) [27], mechanism of injury, Charlson Comorbidity Index [28], American Society of Anesthesiologists (ASA) Score [29], hemodynamic instability due to pelvic injury, initial radiographic assessment, pre- and postoperative measurement anterior and posterior fracture dislocation (mm) as measured in CT radiography of the pelvis, type and timing of initial and definitive surgery, in-hospital complication rates, time to postoperative mobilization to standing or walking (days), and pain at discharge (visual analogue scale [VAS], 0–10).

### Outcomes and definitions

Functional recovery and quality of life were defined as the primary outcomes of this study. These outcomes were assessed in patients who completed the cross-sectional follow-up evaluation using the disease-specific Pelvic Outcome Score (POS) and the EuroQol EQ-5D-5L. The POS comprises three domains: radiographic outcome, clinical outcome, and social reintegration (return to previous employment and/or preinjury levels of sports and social activities). It combines objective findings, such as radiographic fracture reduction, with subjective measures including pain and social reintegration, thereby providing a comprehensive assessment of the patient's functional recovery and residual disability [30]. Based on the POS, outcomes are classified as excellent (7 points), good (6 points), fair (4–5 points), or poor (< 4 points) according to the combined clinical and radiographic scores; the social reintegration domain is not included in this classification [30]. The POS was selected because it integrates radiographic, clinical, and social dimensions of recovery into a single outcome measure. This was considered particularly appropriate for the present study because radiographic fracture reduction was a predefined secondary outcome, allowing evaluation of the relationship between radiographic and patient-reported outcomes within the same assessment framework.

Quality of life was assessed in patients who completed the cross-sectional follow-up evaluation using the EuroQol EQ-5D-5L questionnaire (registration number 60530). The EQ-5D-5L evaluates five domains: mobility, self-care, usual activities, pain/discomfort, and anxiety/depression, each rated on a five-level scale ranging from 1 (no problems) to 5 (extreme problems or inability). In addition, participants rated their overall health at the time of questionnaire completion using the EQ Visual Analog Scale (EQ VAS), with scores ranging from 0 (worst imaginable health) to 100 (best imaginable health) [31].

Secondary outcomes included in-hospital mortality, quality of radiographic fracture reduction according to the radiographic component of the Pelvic Outcome Score [30], length of hospital stay, time to mobilization, pain at discharge and follow-up as measured by the visual analog scale (VAS), and the rates of general and implant-related complications.

The maximum displacement of the anterior and posterior pelvic ring was measured on preoperative and postoperative imaging. The degree of fracture reduction was calculated as the difference between the preoperative and postoperative displacement measurements, representing the correction achieved through surgical intervention. Radiographic measurements were performed independently by the first (TW) and senior (EB) authors. The reviewers were not blinded to treatment allocation. In cases of discrepant measurements, a senior pelvic surgeon and co-author (TB) adjudicated the findings, and the final measurement was established by consensus.

### Statistical analysis

Normality of continuous variables was assessed using the Shapiro–Wilk test and Q–Q plots. As none of the continuous variables were normally distributed, data are presented as medians with interquartile ranges (IQR). Categorical variables are reported as frequencies and percentages. Comparisons between independent groups for continuous variables were performed using the nonparametric Mann–Whitney U test, whereas categorical variables were compared using the chi-square test. Missing data were not imputed and were handled using pairwise exclusion, with missing values omitted only from the respective analyses. All statistical analyses and graphical representations were generated using IBM SPSS Statistics for Windows (Version 29.0; IBM Corp., Armonk, NY, USA). Statistical significance was defined as a two-sided *p* value < 0.05.

## Results

### Baseline characteristics

A total of 158 patients were included in the study. Most were male (*n* = 102, 64.6%), with a median age of 42 years (IQR, 25–59). Overall, 31.0% (*n* = 49) underwent definitive posterior-only (PO) stabilization, whereas 69.0% (*n* = 109) underwent combined anterior–posterior (AP) stabilization (Table 1). The most common mechanism of injury was road traffic accidents (*n* = 93, 58.9%), followed by falls from a height greater than three m (*n* = 54, 34.2%) (Table 1). Stratified analyses of baseline characteristics by ISS, AO/OTA classification, and age are provided in the Supplementary Tables.

**Table 1:** Baseline characteristics

	PO (*N* = 49)	AP (*N* = 109)	p-value
Sex (N,%) Male Female	31 (63.3) 18 (36.7)	71 (65.1) 38 (34.9)	0.63
Age (years) (median, IQR)	35 (21–50)	44 (26–63)	0.17
Injury mechanism (N,%) Road traffic accident Fall < 3 m Fall > 3 m Burial trauma	23 (46.9) 1 (2) 25 (51) 0	69 (63.3) 11 (10.1) 28 (25.7) 1 (0.9)	0.10
ISS (median, IQR)	22 (17–27)	22 (17–26)	0.68
ASA (median, IQR)	3 (3–4)	3 (3–4)	0.54
Charlson Comorbidity Index (median, IQR)	0 (0–1)	0 (0–3)	0.30
Fracture type (N,%) AO/OTA Type B AO/OTA Type C	37 (75.5) 12 (24.5)	60 (55.0) 49 (45.0)	** < 0.05**

*PO* posterior-only stabilization, *AP* combined anterior–posterior stabilization, *ISS* injury severity score, *ASA* American society of anesthesiologists, *AO* arbeitsgemeinschaft für osteosynthesefragen, *OTA* orthopaedic trauma association, *IQR* interquartile range, *N* number

### Primary in-hospital course of treatment

Overall, 8.2% of patients (*n* = 13) died during hospitalization. Traumatic brain injury was the leading cause of death (*n* = 4, 2.5%), followed by multiorgan failure (*n* = 3, 1.9%). Deceased patients had a significantly higher ISS than survivors (median ISS, 38 vs. 22; *p* < 0.05).

Consistent with the significantly higher proportion of AO/OTA type C injuries, pelvic injury-related hemodynamic instability on admission was significantly more common in the AP group (Table 2). Emergency surgery was performed in 113 patients (71.5%), most commonly consisting of supra-acetabular external fixation, followed by sacroiliac screw fixation. For definitive treatment, unilateral sacroiliac screw fixation was the most frequently performed procedure in both groups, followed by transiliac-transsacral screw fixation in the PO group and open reduction and internal fixation (ORIF) of the anterior pelvic ring with plate osteosynthesis in the AP group (Table 2).

**Table 2:** Primary in-hospital course of treatment [all cases]

	PO (*N* = 49)	AP (*N* = 109)	p-value
Time interval till definitive osteosynthetic treatment (days) (median, IQR)	4 (0–8)	3 (0–7)	0.47
Pelvic injury related hemodynamic instability at admission (N, %)	2 (4.1)	33 (30.3)	** < 0.05**
LOS (days) (median, IQR)	21 (12—30)	19 (9–29)	0.46
ICU LOS (days) (median, IQR)	6 (0–14)	6 (0–13)	0.77
Emergent operative treatment (N, %): Pelvic C-clamp External supraacetabular fixation Internal supraacetabular fixation Sacroiliac screw Transiliac transsacral screw ORIF posterior pelvic ring (plate osteosynthesis) ORIF anterior pelvic ring (plate osteosynthesis) CRIF anterior pelvic ring (antegrade/retrograde pubic ramus screw) Lumbopelvic stabilization	0 6 (12.2) 0 13 (26.5) 3 (6.1) 4 (8.2) 0 0 3 (6.1)	8 (7.3) 65 (59.6) 8 (7.3) 38 (34.9) 7 (6.4) 2 (1.8) 10 (9.2) 5 (4.6) 0	0.05 ** < 0.05** 0.05 0.30 0.94 ** < 0.05** 0.44 ** < 0.05** ** < 0.05**
Definitive operative treatment (N, %): External supraacetabular fixation Internal supraacetabular fixation Sacroiliac screw Transiliac transsacral screw ORIF posterior pelvic ring (plate osteosynthesis) ORIF anterior pelvic ring (plate osteosynthesis) CRIF anterior pelvic ring (antegrade/retrograde pubic ramus screw) Lumbopelvic stabilization	0 0 38 (77.6) 10 (20.4) 9 (18.4) 0 (0) 0 (0) 6 (12.2)	24 (22) 24 (22) 76 (69.7) 20 (18.3) 9 (8.3) 46 (42.2) 25 (22.9) 5 (4.6)	** < 0.05** ** < 0.05** 0.31 0.76 0.06 ** < 0.05** ** < 0.05** 0.08

*PO* posterior-only stabilization, *AP* combined anterior–posterior stabilization, *LOS* length of stay, ICU intensive care unit, *CRIF* closed reduction and internal fixation, *ORIF* open reduction and internal fixation, *IQR* interquartile range, *N* number

Despite differences in the osteosynthetic procedures performed, the study groups did not differ significantly with respect to time to surgery, length of hospital or ICU stay, or time to mobilization. Furthermore, no significant differences were observed in the rates of intraoperative or postoperative complications. Overall, AP stabilization was associated with more robust radiographic reduction of the anterior pelvic ring (Tables 3, 4 and 5). Stratified analyses of the primary in-hospital outcomes by ISS, AO/OTA classification, and age are provided in the Supplementary Tables.

**Table 3:** Clinical outcome and follow-up [all cases]

	PO (*N* = 49)	AP (*N* = 109)	p-value
Time to mobilization (standing) (days) (median, IQR)	5 (2–9)	5 (1–9)	0.73
Time to mobilization (walking) (days) (median, IQR)	7 (4–11)	6 (0–15)	0.96
Anterior extent of reduction (mm) (median, IQR)	0 (0–0.5)	4 (0–8.5)	** < 0.05**
Posterior extent of reduction (mm) (median, IQR)	3 (1.25–4.75)	4 (1.75–6.25)	0.18
State of overall health as measured by EQ-5D-5L (VAS) (median, IQR)	67.5 (44.5–80.5)	65 (52.5–77.5)	0.6
POS (overall) (median, IQR)	7 (5–9)	6 (4–8)	0.33
Pain (VAS) (median, IQR)	2 (0–4)	4 (2–6)	** < 0.05**

*PO* posterior-only stabilization, *AP* combined anterior–posterior stabilization, *POS* pelvic outcome score, *VAS* visual analogue scale, *IQR* interquartile range, *N* number

**Table 4:** Clinical outcome and follow-up [AO/OTA Type B]

	PO (*N* = 37)	AP (*N* = 60)	p-value
Time to mobilization (standing) (days) (median, IQR)	4 (1–7)	4 (1–7)	0.88
Time to mobilization (walking) (days) (median, IQR)	7 (4–10)	4.5 (0–10)	0.66
Anterior extent of reduction (mm) (median, IQR)	0	2 (0–6)	** < 0.05**
Posterior extent of reduction (mm) (median, IQR)	2 (1–4)	2 (0–4)	0.65
State of overall health as measured by EQ-5D-5L (VAS) (median, IQR)	75 (56–94)	55 (41–69)	0.12
POS (overall) (median, IQR)	7.5 (6–9)	6 (5–7)	0.09
Pain (VAS) (median, IQR)	2 (0–5)	4 (2–6)	** < 0.05**

*PO* posterior-only stabilization, *AP* combined anterior–posterior stabilization, *POS* pelvic outcome score, *AO* arbeitsgemeinschaft für osteosynthesefragen, *OTA* orthopaedic trauma association, *VAS* visual analogue scale, *IQR* interquartile range, *N* number

**Table 5:** Clinical outcome and follow-up [AO/OTA Type C]

	PO (*N* = 12)	AP (*N* = 49)	p-value
Time to mobilization (standing) (days) (median, IQR)	8.5 (4–14)	8.5 (3–15)	0.73
Time to mobilization (walking) (days) (median, IQR)	9 (5–13)	7.5 (0–19)	0.76
Anterior extent of reduction (mm) (median, IQR)	0 (0–4.63)	6 (0–14.25)	** < 0.05**
Posterior extent of reduction (mm) (median, IQR)	5.5 (2.75–8.25)	5 (2.38–7.63)	0.92
State of overall health as measured by EQ-5D-5L (VAS) (median, IQR)	47.5 (19–76)	70 (60–80)	0.20
POS (overall) (median, IQR)	4 (3–6)	6 (5–7)	0.25
Pain (VAS) (median, IQR)	2 (1–4)	4 (2–7)	0.45

*PO* posterior-only stabilization, *AP* combined anterior–posterior stabilization, *POS* pelvic outcome score, *AO* arbeitsgemeinschaft für osteosynthesefragen, *OTA* orthopaedic trauma association, *VAS* visual analogue scale, *IQR* interquartile range, *N* number

### Follow-up outcomes

A total of 36.7% of patients (*n* = 58) completed the cross-sectional follow-up assessment after a median follow-up of four years (IQR, 7): 44.9% (*n* = 22) in the PO group and 33.0% (*n* = 36) in the AP group. Pain at follow-up, as measured by the visual analog scale (VAS), differed significantly between the groups (median VAS, 2 in the PO group vs. 4 in the AP group; *p* < 0.05). This difference was primarily observed in patients with AO/OTA type B injuries, whereas no significant difference was found among those with type C injuries (Tables 3, 4 and 5).

No significant differences were observed between the groups in the Pelvic Outcome Score (POS), including its radiographic, clinical, and social reintegration domains. Both groups achieved comparable overall POS ratings, corresponding to a fair outcome (median POS, 5 in the PO group vs. 4 in the AP group; *p* = 0.33). Likewise, no significant between-group differences were identified in quality of life, as assessed by the EQ-5D-5L domains of mobility, self-care, usual activities, pain/discomfort, and anxiety/depression. Compared with normative data from the German population, participants in both groups reported slightly greater impairment in the domains of mobility, usual activities, and anxiety/depression (Tables 6 and 7) [32]. Baseline characteristics of follow-up responders and nonresponders were comparable (Table 8). Stratified analyses of follow-up outcomes by Injury Severity Score (ISS), AO/OTA classification, and age are provided in the Supplementary Tables.

**Table 6:** Pelvic Outcome Score

Pelvic Outcome Score (POS)			*p*-value
	PO (*N* = 22)	AP (*N* = 36)	
POS radiological result (median, IQR)	2 (1–3)	2 (1–3)	0.32
POS clinical outcome (median, IQR)	3 (2–4)	2 (1 −3)	0.18
POS overall clinical and radiological score	5 (4–6)	4 (3–6)	0.32
POS social reintegration (median, IQR)	2 (2–3)	2 (2–3)	0.83

*PO* posterior-only stabilization, *AP* combined anterior–posterior stabilization, *IQR* interquartile range, *N* number

**Table 7:** EQ-5D-5L

EQ-5D-5L			*p*-value	German average [32]
	PO (*N* = 22)	AP (*N* = 36)		
Mobility (median, IQR)	2 (2–3)	2 (2–3)	0.97	1
Self-care (median, IQR)	1 (1)	1 (1–2)	0.51	1
Usual activities (median, IQR)	2 (2–3)	2 (2–3)	0.74	1
Pain/discomfort (median, IQR)	2 (1–3)	2 (2–3)	0.36	2
Anxiety/Depression (median, IQR)	2 (1–3)	2 (1–3)	0.87	1

*PO* posterior-only stabilization, *AP* combined anterior–posterior stabilization, *VAS* visual analogue scale, *IQR* interquartile range, *N* number

**Table 8:** Cohort characteristics of follow-up responders and nonresponders

	Responder (*N* = 58)	Nonresponder (*N* = 100)	p-value
Sex (N,%) Male Female	40 (69) 18 (31)	62 (62) 38 (38)	0.40
Age, years (median, IQR)	46 (29–63)	41.5 (26–57)	0.69
Injury mechanism (N,%) Road traffic accident Fall < 3 m Fall > 3 m Burial trauma	39 (67.2) 5 (8.6) 14 (24.1) 0	53 (53) 7 (7) 39 (39) 1 (1)	0.24
ISS (median, IQR)	22 (17–28)	22 (18–27)	0.46
ASA (median, IQR)	3 (2–3)	3 (2—3)	0.47
Charlson Comorbidity Index (median, IQR)	1 (0–3)	0 (0- 2)	
Fracture type (N,%) AO/OTA Type B AO/OTA Type C	34 (58.6) 24 (41.4)	63 (63) 37 (37)	0.61
Time interval till definitive osteosynthetic treatment (days) (median, IQR)	3.5 (0–7)	3 (0—7)	0.17
Pelvic injury related hemodynamic instability at admission (N, %)	11 (19)	24 (24)	0.55
LOS (days) (median, IQR)	21.5 (10—33)	17.5 (10–26)	0.07
ICU LOS (days) (median, IQR)	5 (0–17)	6 (0—10)	0.74
Emergent operative treatment (N, %): Pelvic C-clamp External supraacetabular fixation Internal supraacetabular fixation Sacroiliac screw Transiliac transsacral screw ORIF posterior pelvic ring (plate osteosynthesis) ORIF anterior pelvic ring (plate osteosynthesis) CRIF anterior pelvic ring (antegrade/retrograde pubic ramus screw) Lumbopelvic stabilization	2 (3.4) 25 (43.1) 3 (5.2) 17 (29.3) 5 (8.6) 3 (5.2) 4 (6.9) 2 (3.4) 3 (5.2)	6 (6) 46 (46) 5 (5) 34 (34) 5 (5) 3 (3) 6 (6) 4 (4) 0	0.71 0.74 0.94 0.60 0.38 0.67 0.82 1.0 ** < 0.05**
Definitive operative treatment (N, %): External supraacetabular fixation Internal supraacetabular fixation Sacroiliac screw Transiliac transsacral screw ORIF posterior pelvic ring (plate osteosynthesis) ORIF anterior pelvic ring (plate osteosynthesis) CRIF anterior pelvic ring (antegrade/retrograde pubic ramus screw) Lumbopelvic stabilization	7 (12.1) 6 (10.3) 41 (70.7) 16 (27,6) 7 (12.1) 16 (27.6) 10 (17.2) 8 (13.8)	17 (17) 18 (18) 73 (73) 14 (14) 11 (11) 30 (30) 15 (15) 3 (3)	0.49 0.25 0.85 0.06 0.84 0.86 0.82 ** < 0.05**
Time to mobilization (standing) (days) (median, IQR)	5 (0–8)	5 (0–9.5)	0.93
Time to mobilization (walking) (days) (median, IQR)	7 (2–13)	6.5 (1—13)	0.67
Anterior extent of reduction (mm) (median, IQR)	2 (0–8)	2 (0—6)	0.58
Posterior extent of reduction (mm) (median, IQR)	4 (1–7)	3 (0—5)	0.49

*AP* combined anterior–posterior stabilization, *PO* posterior-only stabilization, *AO* arbeitsgemeinschaft für osteosynthesefragen, *OTA* orthopaedic trauma association, *ORIF* open reduction and internal fixation, *CRIF* closed reduction and internal fixation, *ICU* intensive care unit, *LOS* length of stay, *IQR* interquartile range, *N* = number

## Discussion

The optimal strategy for definitive operative stabilization of unstable pelvic ring fractures in polytraumatized patients remains a subject of ongoing debate [6, 8, 10, 33, 34]. The present study compared the outcomes of posterior-only (PO) and combined anterior–posterior (AP) stabilization in polytraumatized patients treated according to an institutional treatment protocol and yielded the following key findings:

1. AP stabilization was used more frequently for mechanically more unstable pelvic ring fractures, in accordance with the institutional treatment protocol, and was associated with more robust radiologic fracture reduction than PO.
2. When applied according to the protocol-driven treatment algorithm, AP and PO stabilization were associated with comparable functional outcomes and quality of life, despite AP being used predominantly in patients with more severe pelvic injuries.
3. No significant differences were observed between the PO and AP groups with respect to length of hospital stay or complication rates.

### Primary in-hospital course of treatment

To our knowledge, no previous study has specifically evaluated whether a protocol-driven decision between combined anterior–posterior (AP) stabilization and posterior-only (PO) fixation influences in-hospital complications in polytraumatized patients with unstable pelvic ring fractures. In the present study, the rate of general and implant-related complications did not differ significantly between the two treatment groups.

Frane et al. analyzed nearly 20,000 open pelvic ring fractures to identify risk factors associated with complications in this patient population. Older age, higher Injury Severity Score (ISS), lower Glasgow Coma Scale (GCS) scores, and hypotension were identified as relevant predictors of complications; however, the influence of the osteosynthetic strategy was not assessed [11]. Similarly, Buller et al. reported in a retrospective cohort study that internal fixation was associated with lower complication rates, whereas external fixation was associated with increased mortality [5]. Cheng et al. suggested that prolonged operative time may represent an independent risk factor for increased mortality and complication rates and could therefore constitute a potential disadvantage of AP stabilization [35].

Although the present study demonstrates that the longer and potentially more complex procedures associated with AP stabilization did not result in higher complication rates or inferior in-hospital outcomes compared with PO fixation, the indication for AP stabilization should remain based on the specific injury pattern and patient characteristics. The potential disadvantages of increased surgical duration and complexity must be carefully balanced against the potential benefit of improved radiographic reduction. Overall, AP stabilization demonstrated comparable complication rates and length of hospital stay compared with PO fixation, despite being more frequently applied in patients with more unstable type C injuries.

### Impact of radiologic fracture reduction

AP stabilization was associated with more robust radiologic fracture reduction, particularly when applied to more unstable pelvic ring injury patterns according to the institutional treatment protocol. Although restoration of pelvic ring symmetry was achieved in our cohort without an increase in perioperative complications, this potential advantage of AP stabilization must be interpreted in the context of the increased surgical duration and invasiveness associated with this approach, particularly in the vulnerable polytraumatized patient population.

Lai et al. demonstrated an association between inadequate pelvic ring reduction and unfavourable short- and mid-term functional outcomes [36]. n contrast, Dienstknecht et al. reported poorer functional outcomes in patients with posterior or anterior–posterior pelvic ring injuries compared with those with isolated anterior injuries [37]. Thus, the influence of anterior ring injury and its anatomical restoration on patient-reported outcomes remains unclear. Despite its beneficial effect on restoration of pelvic symmetry, the present study did not demonstrate superior long-term patient-reported outcomes following AP stabilization. These findings suggest that radiographic restoration of pelvic ring anatomy does not necessarily translate directly into improved functional recovery.

### Follow-up outcomes

Pain at follow-up was significantly lower in patients treated with PO stabilization. This finding may be related to the higher complexity of fractures, greater soft-tissue injury, and increased burden of concomitant injuries observed in the AP group. This interpretation is supported by Holmes et al., who identified injury severity as a predictor of chronic pain following trauma [38].

In the subgroup analysis stratified by injury type, the significant difference in pain levels was observed only among patients with AO/OTA type B injuries. In contrast, the extensive disruption of the pelvic ring in type C fractures, with subsequent pelvic floor scarring and potential development of refractory chronic pain, may reduce the independent influence of the fixation strategy on long-term pain outcomes [9]. Although patients in the AP group reported significantly higher pain intensity at follow-up compared with the PO group, this difference was not accompanied by impaired quality of life according to the EQ-5D-5L, thereby limiting the clinical relevance of the observed pain difference.

Regarding quality of life after pelvic trauma, the working group “Quality of Life After Pelvic Fractures” and Holstein et al. identified older age, complex trauma, and the overall need for surgical intervention as independent factors associated with poorer posttraumatic quality of life. Furthermore, they reported slightly lower EQ-5D scores among pelvic trauma survivors compared with the general population, which is consistent with the findings of the present study [39]. Gabbe et al. demonstrated that the type of operative treatment, categorized according to anterior or posterior element fixation, was not a major determinant of return to work or independent living [12].

The present study supports these findings using the Pelvic Outcome Score, while focusing not on individual surgical techniques but on the overall stabilization strategy of the pelvic ring (posterior-only versus combined anterior–posterior stabilization). Taken together, these results suggest that the parameters incorporated into our institutional treatment protocol provide an appropriate framework for guiding the selection between AP and PO stabilization.

The findings of the present study suggest that a standardized, protocol-driven approach to definitive pelvic ring stabilization may facilitate consistent clinical outcomes among patients with varying injury severity. Although combined anterior–posterior stabilization was predominantly performed in mechanically more unstable injuries, functional outcomes, quality of life, and complication rates were comparable to those observed after posterior-only fixation. These findings support an individualized treatment strategy based on fracture morphology, patient physiology, and overall trauma burden rather than the routine application of a single stabilization approach.

An important consideration when interpreting these findings is that definitive treatment allocation was determined according to a predefined institutional algorithm rather than randomization. Consequently, patients undergoing combined anterior–posterior stabilization had more severe pelvic injuries, as reflected by the higher proportion of AO/OTA type C fractures and pelvic injury-related haemodynamic instability. The comparable clinical outcomes observed between treatment groups should therefore be interpreted within the context of this protocol-driven treatment selection and may reflect the effectiveness of individualized management rather than direct equivalence of the fixation strategies themselves. This represents an example of potential confounding by indication in observational comparative effectiveness research and should be considered when interpreting the present findings [40].

### Strengths and limitations

To our knowledge, this study is the first to compare PO and AP stabilization as treatment strategies for pelvic ring fractures in a cohort of polytraumatized patients treated according to a predefined institutional treatment protocol. This approach enabled comparison of clinically relevant treatment groups within a standardized decision-making framework. Patients were treated at a level I trauma center by a dedicated and experienced pelvic surgery team. Furthermore, the follow-up assessment consisted primarily of patient-reported outcomes, which represent clinically meaningful measures from a patient-centered perspective. With a predominantly male population and a median age of approximately 40 years, the study cohort is comparable to previously reported populations of polytraumatized patients with pelvic ring fractures. Other baseline characteristics, including mortality rates and injury mechanisms, also appear to be consistent with findings reported in the existing literature [1, 3, 4, 41–43].

Nevertheless, the present study has several limitations. First, its generalizability is limited by its retrospective design and single-centre setting. Treatment allocation followed the institutional treatment algorithm and was therefore based on fracture morphology and physiological status rather than randomization, introducing the potential for confounding by indication. Although subgroup analyses stratified by AO/OTA fracture type, age, and injury severity demonstrated consistent findings, residual confounding from measured and unmeasured variables cannot be excluded. Missing data were handled by pairwise exclusion during group comparisons; however, their presence may have reduced the statistical power of the analyses, which were additionally influenced by the relatively small sample size. The follow-up rate was limited, with only 37% of patients completing the follow-up assessment, potentially introducing a risk of follow-up bias. This may have resulted from missing or incorrect contact information, severe psychiatric comorbidities, or patients being unreachable due to residence outside the country. However, baseline characteristics of follow-up responders and nonresponders were comparable, suggesting that the follow-up cohort was representative of the overall study population. Finally, due to the cross-sectional design of the follow-up assessment, the duration of follow-up varied between patients, although the mean follow-up duration was comparable between treatment groups.

## Conclusions

In conclusion, this study demonstrates that combined anterior–posterior stabilization (AP), when applied according to a structured institutional protocol, is associated with comparable outcomes to posterior-only stabilization (PO) in polytraumatized patients with unstable pelvic ring fractures. Despite being predominantly used for injuries with greater mechanical instability, AP stabilization did not result in higher complication rates, longer hospital stays, or inferior functional outcomes compared with PO fixation. Although AP was associated with greater radiographic fracture reduction, this advantage did not translate into superior long-term patient-reported outcomes, highlighting the complex relationship between radiographic restoration of pelvic symmetry and functional recovery. Lower pain levels at follow-up were observed in the PO group, particularly among patients with AO/OTA type B injuries; however, this difference was not associated with clinically relevant impairment in overall quality of life. These findings further emphasize that the overall burden of polytrauma and injury severity are important determinants of long-term outcomes. Therefore, the selection of a stabilization strategy should be individualized according to pelvic injury morphology, patient physiology, and overall trauma burden within the framework of Early Appropriate Care principles. While the findings support the utility of a standardized, differentiated surgical protocol, prospective multicenter studies with larger cohorts and standardized follow-up assessments are required to validate these results and further elucidate factors influencing long-term functional recovery in this patient population.

## Supplementary information

Below is the link to the electronic supplementary material.

Supplementary file1 (DOCX 30 KB)

## Data Availability

The data collected will be stored securely at our institute for 10 years. During this period, they are still available upon request. After 10 years, the data will be deleted, however, all the datasets analyzed or generated during this study will be available from the corresponding author upon reasonable request.
