# Supplementary material for: Anterior–posterior versus posterior-only stabilization of unstable pelvic ring injuries in polytraumatized patients: a comparative analysis of clinical, radiological, and long-term functional outcomes
Source: Int Orthop. 2026 Jul 20;50(11):2977–87. doi: 10.1007/s00264-026-06966-1 (PMC13694505; doi:10.1007/s00264-026-06966-1)
Supplement: Supplementary file 1 [file 264_2026_6966_MOESM1_ESM.docx]

**Supplementary Table 1:** Baseline characteristics, primary in-hospital course of treatment and outcomes stratified by Injury Severity Score

|  | ISS 16 – 24 (N=102) | ISS > 24 (N=56) | p-value |
| --- | --- | --- | --- |
| Sex (N,%)  Male  Female | 67 (65.7)  35 (34.3) | 35 (62.5)  21 (37.5) | 0.73 |
| Age (years) (median, IQR) | 46.5 (28.5 – 64.5) | 35 (22 – 48) | **<0.05** |
| Injury mechanism (N,%)  Road traffic accident  Fall < 3m  Fall > 3m  Burial trauma | 59 (57.8)  12 (11.8)  31 (30.4)  0 | 33 (58.9)  0  22 (39.3)  1 (1.8) | 0.38 |
| ASA (median, IQR) | 3 (2 – 3) | 3 (2 – 3) | 0.82 |
| Charlson Comorbidity Index (median, IQR) | 0 (0 – 3) | 0 (0 – 1) | 0.51 |
| Fracture type (N,%)  AO/OTA Type B  AO/OTA Type C | 71 (69.9)  31 (30.4) | 26 (46.4)  30 (53.6) | **<0.05** |
| Time interval till definitive osteosynthetic treatment (days) (median, IQR) | 3 (0 – 6) | 2 (0 – 8) | 0.55 |
| Pelvic injury related hemodynamic instability at admission (N, %) | 16 (15.7) | 19 (33.4) | **<0.05** |
| LOS (days) (median, IQR) | 18 (10 – 26) | 22 (16 – 27) | 0.13 |
| ICU LOS (days) (median, IQR) | 4 (0 – 10) | 11 (2 – 20) | **<0.05** |
| Emergent operative treatment (N, %):  Pelvic C-clamp  External supraacetabular fixation  Internal supraacetabular fixation  Sacroiliac screw  Transiliac transsacral screw  ORIF posterior pelvic ring (plate osteosynthesis)  ORIF anterior pelvic ring (plate osteosynthesis)  CRIF anterior pelvic ring (antegrade/retrograde pubic ramus screw)  Lumbopelvic stabilization | 3 (2.9)  41 (40.2)  4 (3.9)  35 (34.3)  7 (6.9)  3 (2.9)  9 (8.8)  17 (16.7)  5 (4.9) | 5 (8.9)  30 (53.6)  4 (7.1)  16 (28.6)  3 (5.4)  3 (5.4)  1 (1.8)  3 (5.4)  2 (3.6) | 0.13  0.13  0.45  0.48  0.71  0.67  0.1  0.67  0.29 |
| Definitive operative treatment (N, %):  External supraacetabular fixation  Internal supraacetabular fixation  Sacroiliac screw  Transiliac transsacral screw  ORIF posterior pelvic ring (plate osteosynthesis)  ORIF anterior pelvic ring (plate osteosynthesis)  CRIF anterior pelvic ring (antegrade/retrograde pubic ramus screw)  Lumbopelvic stabilization | 10 (9.8)  17 (16.7)  78 (76.5)  17 (16.7)  13 (12.7)  36 (35.3)  17 (16.7)  5 (4.9) | 14 (25)  7 (12.5)  36 (64.3)  13 (23.2)  5 (8.9)  10 (17.9)  8 (14.3)  6 (10.7) | **<0.05**  0.64  0.14  0.4  0.6  **<0.05**  0.82  0.2 |
| Time to mobilization (standing) (days) (median, IQR) | 4 (0 – 7) | 7 (0 – 8) | **<0.05** |
| Time to mobilization (walking) (days) (median, IQR) | 4 (0 – 8) | 10 (3 – 17) | **<0.05** |
| Anterior extent of reduction (mm) (median, IQR) | 2 (0 – 7) | 2 (0 – 6) | 0.61 |
| Posterior extent of reduction (mm) (median, IQR) | 3 (0 – 5) | 3 (1 – 5) | 0.71 |
| State of overall health as measured by EQ 5D 5L (VAS) (median, IQR) | 65 (50.5 – 79.5) | 65 (46 – 84) | 0.54 |
| POS (overall) (median, IQR) | 7 (6 – 9) | 5.5 (4 – 7) | 0.1 |
| Pain (VAS) (median, IQR) | 2.5 (0 - 5) | 3 (1 – 4) | 0.41 |

ISS = Injury Severity Score, ASA = American Society of Anesthesiologists, AO = Arbeitsgemeinschaft für Osteosynthesefragen, OTA = Orthopaedic Trauma Association, LOS = length of stay, ICU = intensive care unit, ORIF = open reduction and internal fixation, CRIF = closed reduction and internal fixation, VAS = visual analogue scale, POS = Pelvic Outcome Score, IQR = interquartile range, N = number

**Supplementary Table 2:** Baseline characteristics, primary in-hospital course of treatment and outcomes stratified by AO/OTA classification

|  | AO/OTA Type B (N=97) | AO/OTA Type C (N=61) | p-value |
| --- | --- | --- | --- |
| Sex (N,%)  Male  Female | 59 (60.8)  38 (39.2) | 43 (70.5)  18 (29.5) | 0.24 |
| Age (years) (median, IQR) | 42 (24 – 50) | 42 (36 – 68) | 0.43 |
| Injury mechanism (N,%)  Road traffic accident  Fall < 3m  Fall > 3m  Burial trauma | 61 (62.9)  9 (9.3)  27 (27.8)  0 | 31 (50.8)  3 (4.9)  26 (42.6)  1 (1.6) | 0.35 |
| ISS (median, IQR) | 22 (19 – 26) | 24 (20 - 29) | **<0.05** |
| ASA (median, IQR) | 3 (2 – 3) | 3 (2 – 3) | 0.24 |
| Charlson Comorbidity Index (median, IQR) | 0 (0 – 2) | 1 (0 – 2) | 0.37 |
| Time interval till definitive osteosynthetic treatment (days) (median, IQR) | 4 (0 – 8) | 3 (0 – 7) | 0.41 |
| Pelvic injury related hemodynamic instability at admission (N, %) | 8 (8.2) | 27 (44.3) | **<0.05** |
| LOS (days) (median, IQR) | 18 (14 – 23) | 23 (12 – 34) | **<0.05** |
| ICU LOS (days) (median, IQR) | 4 (1 – 7) | 10.5 (3 – 18) | **<0.05** |
| Emergent operative treatment (N, %):  Pelvic C-clamp  External supraacetabular fixation  Internal supraacetabular fixation  Sacroiliac screw  Transiliac transsacral screw  ORIF posterior pelvic ring (plate osteosynthesis)  ORIF anterior pelvic ring (plate osteosynthesis)  CRIF anterior pelvic ring (antegrade/retrograde pubic ramus screw)  Lumbopelvic stabilization | 0  31 (32)  7 (7.2)  33 (34)  5 (5.2)  0  6 (6.2)  2 (2.1)  1 (1) | 8 (13.1)  40 (65.6)  1(1.6)  18 (29.5)  5 (8.2)  6 (9.8)  4 (6.6)  4 (6.6)  2 (3.3) | **<0.05**  **<0.05**  0.15  0.6  0.51  **<0.05**  0.93  0.21  0.56 |
| Definitive operative treatment (N, %):  External supraacetabular fixation  Internal supraacetabular fixation  Sacroiliac screw  Transiliac transsacral screw  ORIF posterior pelvic ring (plate osteosynthesis)  ORIF anterior pelvic ring (plate osteosynthesis)  CRIF anterior pelvic ring (antegrade/retrograde pubic ramus screw)  Lumbopelvic stabilization | 11 (11.3)  16 (16.5)  69 (71.1)  16 (16.5)  4 (4.1)  22 (22.7)  16 (16.5)  3 (3.1) | 13 (21.3)  13 (21.3)  45 (73.8)  14 (23)  14 (23)  24 (39.3)  9 (14.8)  8 (13.1) | 0.11  0.65  0.86  0.21  **<0.05**  **<0.05**  0.83  **<0.05** |
| Time to mobilization (standing) (days) (median, IQR) | 4 (1 – 7) | 8.5 (3 – 15) | **<0.05** |
| Time to mobilization (walking) (days) (median, IQR) | 6 (2 – 10) | 8 (0 – 16) | 0.14 |
| Anterior extent of reduction (mm) (median, IQR) | 1 (0 – 4) | 5 (0 – 11) | **<0.05** |
| Posterior extent of reduction (mm) (median, IQR) | 2 (1 – 4) | 5 (2 – 7) | **<0.05** |
| State of overall health as measured by EQ 5D 5L (VAS) (median, IQR) | 63.5 (41 – 86) | 70 (60 – 80) | 0.91 |
| POS (overall) (median, IQR) | 7 (6 – 9) | 5.5 (3.5 – 7.5) | 0.2 |
| Pain (VAS) (median, IQR) | 2 (0 – 4) | 3 (1 – 5) | 0.85 |

ISS = Injury Severity Score, ASA = American Society of Anesthesiologists, AO = Arbeitsgemeinschaft für Osteosynthesefragen, OTA = Orthopaedic Trauma Association, LOS = length of stay, ICU = intensive care unit, ORIF = open reduction and internal fixation, CRIF = closed reduction and internal fixation, VAS = visual analogue scale, POS = Pelvic Outcome Score, IQR = interquartile range, N = number

**Supplementary Table 3:** Baseline characteristics, primary in-hospital course of treatment and outcomes stratified by age

|  | Age 18 – 64 years (N=125) | Age > 64 years (N=33) | p-value |
| --- | --- | --- | --- |
| Sex (N,%)  Male  Female | 83 (66.4)  42 (33.6) | 19 (57.6)  14 (42.4) | 0.41 |
| Injury mechanism (N,%)  Road traffic accident  Fall < 3m  Fall > 3m  Burial trauma | 73 (58.4)  3 (2.4)  48 (38.4)  1 (0.8) | 19 (57.6)  9 (27.3)  5 (15.2)  0 | 0,21 |
| ISS (median, IQR) | 22 (17 – 27) | 20 (17 – 22) | 0.06 |
| ASA (median, IQR) | 3 (2 – 3) | 3 (2 – 3) | 0.34 |
| Charlson Comorbidity Index (median, IQR) | 0 (0 – 1) | 4 (3 – 5) | **<0.05** |
| Fracture type (N,%)  AO Type B  AO Type C | 77 (61.6)  48 (38.4) | 20 (60.6)  13 (39.4) | 0.92 |
| Time interval till definitive osteosynthetic treatment (days) (median, IQR) | 3 (0 – 8) | 3 (0 – 7) | 0.61 |
| Pelvic injury related hemodynamic instability at admission (N, %) | 26 (20.8) | 9 (27.3) | 0.48 |
| LOS (days) (median, IQR) | 21 (12 – 30) | 14 (4 – 24) | 0.08 |
| ICU LOS (days) (median, IQR) | 6.5 (0 – 13) | 5 (0 – 10) | 0.13 |
| Emergent operative treatment (N, %):  Pelvic C-clamp  External supraacetabular fixation  Internal supraacetabular fixation  Sacroiliac screw  Transiliac transsacral screw  ORIF posterior pelvic ring (plates osteosynthesis)  ORIF anterior pelvic ring (plate osteosynthesis)  CRIF anterior pelvic ring (antegrade/retrograde pubic ramus screw)  Lumbopelvic stabilization | 6 (4.8)  56 (44.8)  5 (4)  43 (34.4)  7 (5.6)  5 (4)  8 (6.4)  4 (3.2)  3 (2.4) | 2 (6.1)  15 (45.5)  3 (9.1)  8 (24.4)  3 (9.1)  1 (3)  2 (6.1)  2 (6.1)  0 | 0.67  0.95  0.37  0.3  0.44  0.8  0.94  0.61  0.4 |
| Definitive operative treatment (N, %):  External supraacetabular fixation  Internal supraacetabular fixation  Sacroiliac screw  Transiliac transsacral screw  ORIF posterior pelvic ring (plate osteosynthesis)  ORIF anterior pelvic ring (plate osteosynthesis)  CRIF anterior pelvic ring (antegrade/retrograde pubic ramus screw)  Lumbopelvic stabilization | 18 (14.4)  15 (12)  96 (76.8)  21 (16.8)  16 (12.8)  40 (32)  16 (12.8)  10 (8) | 6 (18.2)  9 (27.3)  18 (54.5)  9 (27.3)  2 (6.1)  6 (18.2)  9 (27.3)  1 (3) | 0.59  0.05  **<0.05**  0.21  0.37  0.14  0.06  0.46 |
| Time to mobilization (standing) (days) (median, IQR) | 5 (1 – 9) | 4 (2 – 7) | 0.2 |
| Time to mobilization (walking) (days) (median, IQR) | 7 (1 – 13) | 5 (0 – 11) | 0.32 |
| Anterior extent of reduction (mm) (median, IQR) | 2 (0 – 6) | 3 (0 – 7) | 0.32 |
| Posterior extent of reduction (mm) (median, IQR) | 3 (1 – 6) | 3.5 (1.5 – 5.5) | 0.75 |
| State of overall health as measured by EQ 5D 5L (VAS) (median, IQR) | 65 (45 – 85) | 60 (51 – 69) | 0.37 |
| POS (overall) (median, IQR) | 6 (5 – 8) | 6 (5 – 8) | 0.91 |
| Pain based (VAS) (median, IQR) | 2 (0 – 4) | 3 (1 – 5) | 0.62 |

ISS = Injury Severity Score, ASA = American Society of Anesthesiologists, AO = Arbeitsgemeinschaft für Osteosynthesefragen, OTA = Orthopaedic Trauma Association, LOS = length of stay, ICU = intensive care unit, ORIF = open reduction and internal fixation, CRIF = closed reduction and internal fixation, VAS = visual analogue scale, POS = Pelvic Outcome Score, IQR = interquartile range, N = number
